# Natural Killer Cells in Human Immunodeficiency Virus-1 Infection: Spotlight on the Impact of Human Cytomegalovirus

**DOI:** 10.3389/fimmu.2017.01322

**Published:** 2017-10-17

**Authors:** Dimitra Peppa

**Affiliations:** ^1^Division of Infection and Immunity, University College London, London, United Kingdom; ^2^Nuffield Department of Medicine, University of Oxford, Oxford, United Kingdom

**Keywords:** human immunodeficiency virus, human cytomegalovirus, natural killer cells, NKG2C, CD57, adaptive

## Abstract

Human cytomegalovirus (HCMV) has been closely associated with the human race across evolutionary time. HCMV co-infection is nearly universal in human immunodeficiency virus-1 (HIV-1)-infected individuals and remains an important cofactor in HIV-1 disease progression even in the era of effective antiretroviral treatment. HCMV infection has been shown to have a broad and potent influence on the human immune system and has been linked with the discovery and characterization of adaptive natural killer (NK) cells. Distinct NK-cell subsets, predominately expressing the activating receptor NKG2C and the marker of terminal differentiation CD57, expand in response to HCMV. These NK-cell populations engaged in the long-lasting interaction with HCMV, in addition to characteristic but variable expression of surface receptors, exhibit reduced expression of signaling proteins and transcription factors expressed by canonical NK cells. Broad epigenetic modifications drive the emergence and persistence of HCMV-adapted NK cells that have distinct functional characteristics. NKG2C^+^ NK-cell expansions have been observed in HIV-1 infected patients and other acute and chronic viral infections being systematically associated with HCMV seropositivity. The latter is potentially an important confounding variable in studies focused on the cellular NK-cell receptor repertoire and functional capacity. Here, focusing on HIV-1 infection we review the evidence in favor of “adaptive” changes likely induced by HCMV co-infection in NK-cell subsets. We highlight a number of key questions and how insights into the adaptive behavior of NK cells will inform new strategies exploiting their unique properties in the fight against HIV-1.

## Introduction

Natural killer (NK) cells are a diverse group of innate lymphocytes residing at the crossroads of innate and adaptive immunity (1). Their remarkable effector agility is achieved via expression of a wide array of receptors and integration of signals that are finely attuned to ensure self-tolerance, while permitting effective responses against viral assaults and tumor transformation. In addition to important immunoregulatory functions (2, 3), a number of murine studies support that NK cells can acquire immunological memory similarly to B and T cells (4–7). While antigen-specific NK responses have been documented in mice and more recently in primates (8), clear evidence for NK-cell memory in humans is lacking. The NK-cell compartment in humans displays phenotypic and functional heterogeneity encompassing populations at various stages of maturation with distinct receptor combinations (9–11). In recent years, it has become apparent that variegated expression of inhibitory and activating receptors at the single cell level leads to a more diverse NK-cell repertoire than previously envisaged. Cytometry by time-of-flight has enabled us to profile the healthy human NK-cell repertoire, uncovering between 6,000 and 30,000 unique NK-cell subsets per individual (12). This observed diversity is generated by a combination of factors including genetic contributions (13, 14), along with differentiation in reprogramming in response to local tissue milieu (15) and infections/environmental factors (12). The substantial influence of environmental factors is supported by twin studies demonstrating that non-heritable factors exert a more profound and cumulative influence compared to heritable traits (16, 17). One such factor is human cytomegalovirus (HCMV), a widespread β-herpesvirus with a prevalence ranging from 40 to 100% depending on age, socioeconomic factors, and geographical region (18). In immunocompetent hosts, HCMV infection is usually subclinical leading to latency, whereas in immunosuppressed patients, including human immunodeficiency virus-1 (HIV-1)-infected and transplant patients, it remains a significant cause of morbidity and potentially life threatening complications (18). HCMV has a broad impact on immunity (16) and has recently been associated with the expansion of adaptive or memory-like NK-cell subsets (19, 20).

In the context of HIV infection, HCMV is a highly prevalent (21) and well-recognized opportunistic pathogen responsible for significant morbidity and mortality prior to the introduction of antiretroviral treatment (ART) (22, 23). However, despite the roll-out of effective ART, HCMV remains a significant cofactor in HIV-1 disease progression (24–26), displaying a strong association with systemic inflammation (27, 28), cardiovascular disease (29, 30), reduced immune resilience (31), and immune senescence (27). A recent report has highlighted the role of HCMV replication in intestinal barrier dysfunction in asymptomatic HIV-1 infection and contribution to persistent immune activation (32). It is thus highly relevant to increase our understanding of the complex inter-relationship between HCMV and HIV-1 and of the effects that it bears on the effector immune response. The recent identification of distinct NK-cell subsets with adaptive properties induced by HCMV has raised a number of intriguing questions, including the ability of other viruses to induce them and their physiological relevance in different disease settings. Here, we summarize findings on the molecular signature of HCMV-adapted NK cells and discuss how NK-cell phenotypic and functional features described in HIV-1 infection could partly reflect the immunological fingerprint of HCMV.

## Features of CMV-Adapted NK Cells—Emphasis on HCMV

Evidence from both murine and human studies has demonstrated an important role for NK cells in antiviral defense against herpesviruses, in particular HCMV (33), reinforced by elaborate viral evasion strategies (34).

Although NK cells have been originally described to represent short-lived innate lymphocytes, they can exhibit persistent memory in response to infections. This is best exemplified by mouse CMV (MCMV) infection, where naive NK cells that express Ly49H, recognizing the virally encoded glycoprotein m157, were reported to clonally expand and to subsequently contract forming a pool of long-lived memory cells (6). MCMV-primed memory NK cells mount a robust response upon secondary challenge with enhanced interferon-γ (IFN-γ) secretion and cytotoxicity (6), but display reduced “bystander” functionality to heterologous infection suggesting the specialized nature of these cells (35).

**Figure 1:**
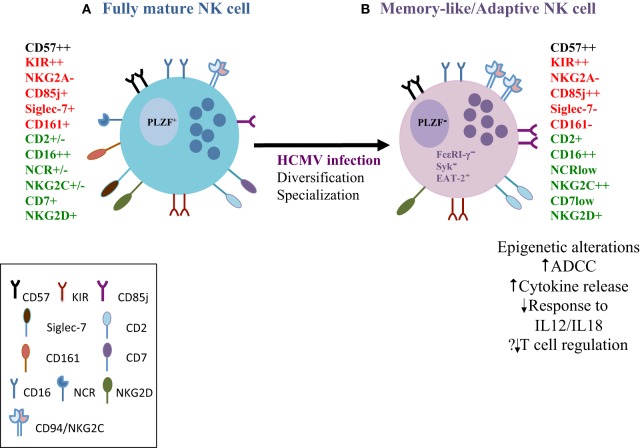
The phenotypic, functional and molecular attributes of human cytomegalovirus (HCMV)-adapted natural killer (NK) cells. **(A)** As CD56dim NK cells go through the spectrum of differentiation they gradually lose expression of the inhibitory receptor NKG2A, natural cytotoxicity receptors and sequentially acquire more specific inhibitory receptors, such as inhibitory killer immunoglobulin receptors (KIRs) and CD85j. KIR acquisition is important in determining the functional fate of the NK cells. CD57 expression represents a terminal step in the differentiation process. Fully mature NK cells gain cytolytic ability and are efficient in mediating antibody-dependent cellular cytotoxicity (ADCC) **(B)** NK cells with adaptive features expanded in response to HCMV infection are distinct from conventional NK cells on the basis of expression of surface receptors, such as high expression of NKG2C, lower expression of the inhibitory Siglec-7, and down-regulation of the transcription factor promyelocytic leukemia zinc finger and key signaling molecules (FcεRI-γ, Syk, and EAT-2). Different combinations of expression patterns result in considerable heterogeneity among adaptive NK cells. Epigenetic diversification leads to altered target cell specificities and functional specialization that includes enhanced ADCC (increased IFN-γ and TNF-α against opsonized HCMV-infected targets) but reduced responsiveness to cytokine stimulation and reduced degranulation against autologous T cells. Red = inhibitory receptors; green = activating receptors.

Congruent with animal models, HCMV infection has been shown to induce an adaptive reconfiguration of the NK-cell compartment. Seminal work by Lopez-Botet’s group described a higher proportion of NK cells expressing the DAP-12 coupled NKG2C receptor in healthy individuals seropositive for HCMV (36, 37). These observations have been extended to hematopoietic stem cell transplantation (38, 39) and solid organ transplantation (40). Expansion of these subpopulations of NK cells and their subsequent longevity resembled clonal expansion of adaptive immune cells. Expanded NKG2C^+^ NK cells display a differentiated phenotype characterized by expression of CD57, increased expression of the inhibitory CD85j (38, 40), and a preferential oligoclonal pattern of inhibitory killer immunoglobulin receptors (KIRs) for self HLA-C1 and/or C2 allotypes (41, 42). In addition, they lack NKG2A, the inhibitory counterpart of NKG2C sharing specificity for HLA-E, and express lower levels of natural cytotoxicity receptors (NCR: NKp30 and NKp46) (36), CD161, CD7, and Siglec-7 (43–45) and have higher expression of CD2 involved in their activation (46, 47). Expression of other receptors such as NKG2D is maintained (36). The phenotypic hallmarks of adaptive NK cells are summarized in Figure 1. Of note, the magnitude of the HCMV imprint on NK-cell subsets varies within seropositive individuals (i.e., the NKG2C^bright^ phenotype is found in 50% of HCMV^+^ individuals) and the adaptive NKG2C^+^ compartment can persist in high frequencies for years (41). Subclinical or tissue specific reactivations of HCMV during latency may contribute to the maintenance of NK^+^NKG2C^+^ pool in addition to NKG2C copy number and age-related changes in NK-cell differentiation (48, 49). The exact ligand involved in recognition and the cellular mechanisms driving the expansion of NKG2C^+^ NK cells are yet to be elucidated. It remains unclear whether this is mediated through interaction with its cellular ligand HLA-E alone, HLA viral loaded peptide or an unknown ligand of host or viral origin (41, 50–52).

The large phenotypic heterogeneity of adaptive NK cells extending beyond the NKG2C^+^ subset, is illustrated by the detection of NK-cell subsets sharing numerous attributes of adaptive NK cells in individuals independent of NKG2C or in the absence of NKG2C (KLRC2-deficient individuals) and in transplant recipients of NKG2C null grafts (41, 47, 53). Strikingly, these HCMV-driven expansions encompass activating KIRs (53), suggesting their potential role in the recognition and response to HCMV.

Further reports described a subset of human NK cells deficient for the adaptor protein FcεRI-γ, which was strongly associated with HCMV seropositivity (54). FcεRI-γ^−^ NK cells share a lot of the characteristics of adaptive NK cells, respond robustly to CD16 stimulation (55) and similar to NKG2C^+^ cells display more vigorous effector responses to HCMV-infected targets, but only in the presence of HCMV-specific antibodies (54, 56). NK cells lacking FcεRI-γ expand in response to HCMV-infected targets accentuated by the presence of anti-HCMV antibody, highlighting the role of specific humoral immunity in also favoring their preferential expansion (57–59). Interestingly, these cells also responded to herpes-simplex virus-1 (HSV-1)-infected targets in the presence of HSV-1 plasma (54) demonstrating cross-protection to other viruses. The enhanced effector function of this subset was attributed to selective and more potent signaling through the CD3ζ chain, which has three immunoreceptor tyrosine-based activation motifs. Subsequently, CD2 has been identified as a key co-stimulatory receptor synergizing with CD16 to stimulate increased cytokine production in adaptive NK cells (47). Global epigenetic profiling has identified commonalities between adaptive NK cells and memory CD8 T cells (58, 60). These adaptive NK cells are marked by DNA methylation silencing of the transcription factor, promyelocytic leukemia zinc finger (PLZF), as well as stochastic down-regulation of several signaling molecules, such as Syk, EAT-2, and DAB-2 (58, 60). PLZF is known to interact with several target genes, including IL12RB2, IL18RAP, and KLRB1 (61), explaining the lack of responsiveness to IL12/IL18 stimulation (58). However, in comparison to conventional NK cells, adaptive NK cells display augmented IFN-γ and TNF-α production when triggered via antibody-dependent cellular cytotoxicity (ADCC); the hypomethylated IFN-γ and tumor necrosis factor (TNF) regulatory regions in adaptive NK cells provide a mechanism for increased cytokine production (58, 60). Interestingly, adaptive NK cells display reduced degranulation toward activated autologous T cells (58), which may impact on the regulation of immune responses.

Taken together, these results suggest the heterogeneity and functional specialization of adaptive NK cells in the immunosurveillance of infected cells and functional bias toward ADCC (Figure 1). Whereas the expansion of adaptive NK cells may serve as a strategy to control HCMV, during its life long interaction with the host, it remains unclear whether other viral infections can induce adaptive properties in NK cells. Although potential cross-reactivity of adaptive NK cells could confer an advantage in the tumor setting such as reduced relapse risk in leukemia patients (62, 63), their role in the control of heterologous infections or post vaccination is less well defined (64, 65).

## Skewing and Adaptation of NK Cells to HIV-1 Infection: The Confounding Effect of HCMV

Accumulating data support an important role for NK cells in the control of HIV-1 infection and protection against disease acquisition (66–68). These stem from elegant genetic studies linking specific KIR/HLA combinations with HIV-1 outcome (66, 67), functional studies where protective KIR alleles are associated with enhanced NK-cell cytolytic function *in vitro* (69) and evidence of KIR-facilitated immune pressure on HIV-1 to escape NK-cell recognition (70). However, chronic HIV-1 infection is known to alter NK-cell composition and effector function. This has been documented by a number of studies with often conflicting results, which can be attributed to a number of factors including the influence of immunogenetics, disease state, and the cross-sectional nature of studies. The latter have not always adequately controlled for a number of confounding factors such as age, gender, ethnicity, and HCMV serostatus among HIV-1-infected and HIV-1-negative controls. Given the high prevalence of HCMV co-infection within HIV cohorts and the profound skewing and adaptation of NK cells to HCMV, this is an important variable to consider when interpreting findings.

HIV-1 viremia is associated with a significant and pathological redistribution of the NK compartment with the emergence of an aberrant CD56^−^CD16^+^ NK-cell subset (71, 72). This rare population displays phenotypic perturbations, including down-regulation of the activating NCRs, and features in common with mature CD56^dim^ NK cells (72, 73). It has been proposed to represent an activated subset generated from chronic target engagement with impaired function. Recent studies have demonstrated that a decreased expression of the c-lectin-type inhibitory receptor, Siglec-7, on NK cells occurs early during HIV-1 infection and precedes the loss of CD56 (74). Expression of Siglec-7 is not affected in long-term non-progressors (LTNP), and ART leads to a progressive restoration of NK-cell subsets (74). Paralleling the observations in HIV-1 infection, HCMV reactivation in patients undergoing umbilical cord blood transplantation has been shown to induce the expansion of the CD56^−^/CD16^+^/Siglec-7^−^ NK-cell subset (38). The expansion of hypofunctional CD56^−^ NK cells following HCMV reactivation likely occurs when T-cell immunity is impaired and may also reflect the modulating effects of HCMV. It remains to be determined whether the CD56^−^/CD16^+^ subset represents a subgroup of NK cells with adaptive features that has become anergic following repeated stimulation.

A number of other studies have reported a variable degree of perturbations in the NK-cell repertoire consistent with a dichotomous effect of viremia, including down-regulation of activating NK-cell receptors and up-regulation of expression of inhibitory NK receptors (iNKRs) (75–77). Collectively, these changes have been described to contribute to defective NK-cell function described in HIV-1 infection (76, 77). Although the HCMV serostatus is not always considered in these studies, it is plausible that these changes are biased by HCMV co-infection and possible reactivation with increasing immunosuppression. Along these lines, the observed down-regulation of NCRs, stable expression of NKG2D, and higher levels of CD85j and skewing of inhibitory KIRs (although not consistently reported) bear phenotypic resemblance to NK-cell subsets with adaptive features described in HCMV infection. NK cells in HIV-1 infection exhibit a higher ratio of CD57^+^ to CD57^−^ due to the loss of CD57^−^ cells in comparison to healthy controls; however, this comparison may be confounded by the HCMV status of these individuals, which was not reported (78). A shift toward a more mature terminally differentiated NK-cell phenotype is nonetheless supported by a study of HIV-1 infected individuals on effective ART, demonstrating that HCMV accelerates age-related increases in CD57 expression (79).

The most convincing evidence of the impact of HCMV co-infection on the NK-cell repertoire in HIV-1 infection comes from reports on NKG2C expression. Guma et al. originally proposed that HCMV co-infection is responsible for the expansions of NKG2C^+^ NK cells encountered in HIV-1 infected individuals (80). These findings were further supported by additional studies when the HCMV serostatus was taken into consideration (81, 82). The dramatic expansion of NKG2C^+^ NK cells in HIV-1 infected individuals was accompanied by a decrease in the expression of NKG2A leading to a low NKG2A/C^+^ NK-cell ratio; these changes were attributed to concomitant infection and/or HCMV reactivation rather than being a consequence of HIV-1 infection alone (82). A number of reports describe NKG2C^+^ NK-cell expansions in several acute and chronic viral infections, being systematically associated with HCMV co-infection (83–86). Although the relative increase in the proportions of NKG2C^+^ NK cells between HIV-1-infected and HIV-1-uninfected HCMV seropositive individuals varies between studies and cohorts (80, 81), the data suggest that the impact of HCMV exposure is potentially greater in HIV-1 infection. It has been suggested by animal models that the differentiation of adaptive NK cells is driven by inflammation (87). Thus, it is plausible that adaptive NK-cell expansions may be inflated in HIV-1 infected individuals, as a result of lack of immune control, ongoing immune activation and higher infectious burdens, including HCMV. One could speculate that the size of the HCMV imprint represents a compensatory mechanism in antiviral defense especially when T-cell-mediated control is impaired (88). It remains uncertain whether HCMV reactivation occurs alongside acute infection or alternatively whether pre-existing HCMV primed NK-cell subsets expand in response to secondary viral infection alone. HIV-1 causes down-regulation of HLA-A, B while retaining HLA-E expression (89, 90), similar to HCMV maintaining/stabilizing HLA-E expression (91, 92). Thus, a direct effect of HIV-1 on NKG2C^+^ NK-cell expansion is conceivable. The recently reported down-regulation of HLA-C by most primary HIV-1 clones (93) raises questions about the ability of HCMV expanded NKG2C^+^ NK cells, preferentially expressing self-HLA-C KIRs, to recognize “missing-self” on HIV-infected targets compared to mature educated NK cells.

Open questions remain regarding not only the mechanism but also the clinical implications of such HCMV-NK-cell interaction in terms of protection against acquisition and HIV-1 disease progression. NKG2C deletions have been linked to a higher risk of contracting HIV-1, in addition to accelerated disease progression and elevated pre-treatment viral load (94). Although these findings are interesting, this study did not report and correct for the influence of HCMV co-infection. One could speculate that the expansion of NKG2C^+^ NK cells in HCMV seropositive individuals may confer protection against primary HIV-1; this notion is however not supported by some older observations that prior infection with HCMV is associated with low CD4 count, progression to AIDS and increased mortality (95). It has been suggested that maturation leads to divergence and increased NK-cell receptor diversity was found to be associated with an increased risk of HIV-1 acquisition in a small cohort of high-risk women (96). Given that viral challenge may increase receptor diversity, further work is required to determine whether this represents reduced plasticity to new challenging pathogens or whether it is linked to other immune characteristics such as exhaustion. Recently, a subpopulation of PD1^+^ NK cells, mainly composed of fully mature NK cells, has been described in HCMV^+^ individuals (97). It would be of interest to assess whether NK cells expanded in HCMV/HIV-1 co-infection succumb to continuous stimulation and examine the factors that may contribute to the induction of PD1 in this setting. PD1 signaling could therefore down-regulate not only T-cell-mediated responses but also innate responses, and this mechanism may be particularly prominent in HIV-1 infection (98).

Conversely, a link between a mature NK-cell compartment (CD57^+^) and decreased levels of viral load and immune activation at the time of the primary HIV-1 infection has been reported. Those patients with a mature NK profile at inclusion showed a better early response to ART in comparison to patients with an immature NK profile (99). However, the HCMV serostatus of these individuals is not recorded and the status of NK cells at the point of infection is not known. Whether mature CD57^+^ or NKG2C^+^CD57^+^ NK cells represent adaptive NK cells that contribute directly to better virus control during acute HIV-1 infection and how their role evolves during chronic infection remain unclear.

In agreement with the findings in HCMV seropositive individuals, an NK-cell population that lacks FcεRI-γ expression and has superior ADCC activity has been identified in HIV-1 viremic individuals and shown to persist following virological suppression with ART (100, 101). This subset shares some phenotypic characteristics with adaptive NK cells induced by HCMV (100). Although this subset is associated with HCMV antibody levels in the general population, in HIV-1-infected individuals correlates with inflammatory markers (100). The long-term effects of expansion of FcεRI-γ-deficient NK cells in HIV-1 infection needs to be further elucidated given a possible role in tumor surveillance. Nonetheless, the identification of a subset with enhanced ADCC activity in HIV-1 infection has potentially important implications for the design of vaccine strategies aimed at generating ADCC-promoting antibody responses.

**Figure 2:**
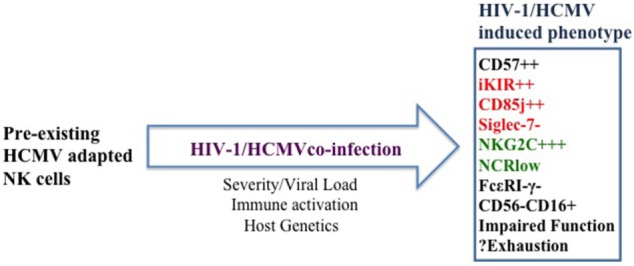
Proposed model of the cumulative effect of human cytomegalovirus (HCMV) and ongoing immune activation on natural killer (NK) cells. Pre-existing HCMV-adapted NK cells expand during human immunodeficiency virus-1 infection to a variable degree depending on the tempo of HCMV reactivation, underlying level of immune activation, decreased T-cell-mediated control, and host genetics. HCMV co-infection accelerates NK-cell maturation and partly underlies the expansion of NK subsets with adaptive features in addition to the emergence of an aberrant CD56^−^CD16^+^ NK-cell subset. Whether these subsets become progressively dysregulated or exhausted remains to be determined.

These collective data demonstrate that a number of the phenotypic NK-cell features described in HIV-1 bear the trademarks of HCMV infection (Figure 2). With increased definition of the assortment of NK-cell subsets with adaptive features driven by HCMV infection and the increased appreciation of HCMV in driving ongoing immune activation even during effective ART, it would be important to reassess the NK-cell repertoire composition, their response potential in different phases of infection and stimulus-dependent functional properties. A comprehensive analysis of the transcriptional signatures and epigenetic modifications of NK cells in HIV-1 infection is lacking and worth exploring.

## Concluding Remarks and Future Perspectives

The potent effector function of NK cells and the rapidity of NK-cell response have identified them as key areas for research. Recent reports about the diversity of NK-cell repertoire and ability to assume adaptive features in response to HCMV infection and even display memory-like responses to cytokines (102) and antigen-specific responses in primates (8) have opened up prospects for the generation of new therapies. HCMV co-infection is highly prevalent in HIV-1 infected cohorts and remains an important cofactor in disease progression even in the era of ART. Both HIV-1 and HCMV as well as immune activation can further shape NK-cell responsiveness and differentiation. It is therefore important to capture the diversity of the NK-cell repertoire and identify potentially novel adaptive signatures of NK-cell subsets with preserved activation pathways. Whereas a number of questions remain regarding the epigenetic diversification, development, and persistence of NK cells with adaptive properties, elucidating how clonal NK-cell populations can be directed or reshaped will critically inform our ability to harness NK cells toward a therapeutic goal.

## Author Contributions

The author confirms being the sole contributor of this work and approved it for publication.

## Conflict of Interest Statement

The author declares that the research was conducted in the absence of any commercial or financial relationships that could be construed as a potential conflict of interest. The reviewer AH and handling editor declared their shared affiliation.
